# A Multicenter, Prospective, Observational, Open-Label Study of the Safety and Comfort of Gensulin^®^ Delivery Device Use in a Large Cohort of Adult and Elderly Patients with Type 2 Diabetes

**DOI:** 10.3390/ijerph17207587

**Published:** 2020-10-19

**Authors:** Małgorzata Masierek, Katarzyna Nabrdalik, Hanna Kwiendacz, Tomasz Sawczyn, Janusz Gumprecht

**Affiliations:** 1 Department of Internal Medicine, Diabetology and Nephrology in Zabrze, Faculty of Medical Sciences in Zabrze, Medical University of Silesia, 41-800 Katowice, Poland; malgorzata.masierek@bioton.com (M.M.); hanna.kwiendacz@gmail.com (H.K.); jgumprecht@sum.edu.pl (J.G.); 2 BIOTON S.A., 02-516 Warszawa, Poland; 3 Department of Physiology in Zabrze, Faculty of Medical Sciences in Zabrze, Medical University of Silesia, 41-800 Katowice, Poland; sawczyn.t@googlemail.com

**Keywords:** diabetes mellitus type 2, insulin pen, safety, satisfaction

## Abstract

Insulin treatment is necessary for many patients with type 2 diabetes, and its delivery must be safe and comfortable. This study evaluated patients’ safety and comfort when using a Gensulin^®^ delivery device, GensuPen (Bioton), a reusable insulin pen device for injecting Gensulin^®^ insulin among adult and elderly patients with type 2 diabetes. This was a 4-week multicenter, prospective, observational, open-label study in patients with diabetes mellitus type 2 who have recently started using a GensuPen. Overall, 10,309 patients (mean age: 63 ± 12.0 years; 47.9% female) were analyzed in this study. Of these, 2.5% had used an insulin delivery device before, and for 97.5%, GensuPen was the first delivery device they had used. Most (87.8%) of the patients rated the GensuPen as very good in setting the dose, 92.0% in confirmation of successful insulin administration, 80.9% in trigger location, and 75.0% in force needed for injection. The overall safety of the GensuPen use was high since severe hypoglycemia occurred only in 0.2% of the studied patients. There were 0.6% adverse events, none of which were serious. This real-life observation data shows that the GensuPen was well accepted and safe in this large patient population of adult and elderly patients with type 2 diabetes.

## 1. Introduction

Four-hundred-sixty-three million adults globally have diabetes, and the prevalence is projected to reach 700 million by 2045 [1]. The vast majority of these suffer from type 2 diabetes (T2DM). The International Diabetes Federation (IDF) estimates that more than 59 million adults with diabetes live in the European region. In Poland, there were 2,344,600 cases of diabetes in 2019, where the total adult population is estimated to be 28,891,100; so, the prevalence in adults in Poland is 8.1%. Even though there are many new non-insulin drugs introduced into the market, insulin treatment is still necessary for life for many patients with T2DM [2]. Basu et al. recently estimated that globally among patients with T2DM, 7.5% were using insulin, which corresponds to around 30 million people, and the same percentage has been observed in Europe [3].

Besides insulin itself, the delivery device is also important. In 1921, insulin was first administrated using glass syringes and a reusable needle. The first manufactured insulin pen, the NovoPen^®^ (Novo Nordisk), was introduced in 1985 [4]. Insulin administration devices have attracted much research interest during the last decade. At that time, the authors published studies proving that a pen injector is a safer way of delivering insulin than a vial-and-syringe. However, this is no longer the case, especially in Europe, where now the majority of patients with T2DM are using pens [5,6]. Before 2010, Gensulin^®^, manufactured by Bioton S.A., Poland, was administered with the universal Autopen device, not dedicated solely to Gensulin^®^. In 2010, Bioton introduced GensuPen, an automatic insulin pen injector approved exclusively for the administration of 3 mL insulin cartridges according to ISO 11608-3 of the recombinant human short- or intermediate-acting insulin (Gensulin R^®^ or Gensulin N^®^ respectively) or a premix human insulin (Gensulin M30^®^, M40^®^, M50^®^). This is a pen with a spring-assisted insulin release, an automatic mechanism, and a lateral trigger. The device is equipped with a colored pointer, which appears when the delivery of insulin is completed successfully (green light). Additionally, turns of the dose selector forwards and backward to adjust the dose are indicated by sounds. It was proven in the study by Fendler et al. [7] that the next generation GensuPen, namely GensuPen 2, significantly reduced the force required for insulin injection compared to HumaPen Ego and NovoPen 4, which can be especially useful for patients with impaired manual dexterity.

Over a dozen different insulin injection pen devices are available in Poland, as each pharmaceutical company produces its own equipment, and they differ in size, mechanism, weight, and colors. The GensuPen 2 differs from GensuPen in just two features: the maximal dose of insulin per injection, which was enlarged from 40 to 60 IU, and a minimum amount of insulin that can be dialed at one time, which was lowered from 2 IU to 1IU. One should remember that as with any other medical technology, safe and proper use is vital. People using insulin injection pen devices acknowledge special convenience related to its use, namely that they can hear and feel adjustments to the dose. They do not have to use much force, which is especially important for patients with reduced manual dexterity and visual impairment [8]. When we take a closer look at the demographics of patients with diabetes in general, it should be further emphasized that assessing safety and comfort with medical devices should receive special attention among the older group of patients. Typically, a person is considered old when they have reached the age ≥ 60 or 65 years [9]. Nowadays, one in five of the people above 65 years old have diabetes [10]. Many insulin manufacturers perform their own post-marketing, real-life studies related to devices dedicated to their produced insulins, and the outcomes are usually based on structured self-prepared questionnaires [11,12,13,14]. Here, we have performed an observational study aiming to assess safety (number of severe hypoglycemic episodes) and patient comfort (structured questionnaire) when using the GensuPen in a large cohort of adult and elderly patients with T2DM.

## 2. Materials and Methods

The “GensuPen in Practical Use” was a 4-week multicenter, prospective, observational, open-label study performed between March 2011 and January 2012. Eligible patients were recruited from outpatient diabetology clinics and diabetology wards distributed all over Poland. Each of the 1172 diabetologists participating in the study recruited approximately ten consecutive patients seen on a regular visit fulfilling the inclusion/exclusion criteria. Due to the study’s observational character, there were no defined study procedures, and the diabetologists performed all assessments as a part of routine clinical care.

Patients were eligible for the study if they had been diagnosed with T2DM at least 12 months before enrollment based on clinical criteria [15], were aged >18 years, were current insulin users who, for a medical reason, had to have the insulin type or manufacturer changed, or insulin naïve patients who were to start an insulin therapy with human insulin. Exclusion criteria included alcohol or narcotic drugs addiction, dementia or mental condition, and allergy to insulin.

The study comprised one site visit at the time of enrollment and two telephone contacts. One telephone contact was performed 7 days following the enrollment into the study. The second telephone contact was performed following 4 weeks ± 7 days from the day of the enrollment into the study. Patients were assessed at the site, and were asked to list potential complaints related to previous pen injectors if applicable. All treatment decisions were made following local clinical practice. All participating patients were maintained on Gensulin (Gensulin R^®^ or Gensulin N^®^, or a premix insulin Gensulin M30^®^, M40^®^, or M50^®^) and were educated regarding the proper use of the GensuPen and the proper insulin injection technique according to routine local practice. Written educational materials were also handed out to the patients. Patients were also advised to contact a dedicated GensuPen telephone help-line in case of any technical problems using the GensuPen.

**Figure 1:**
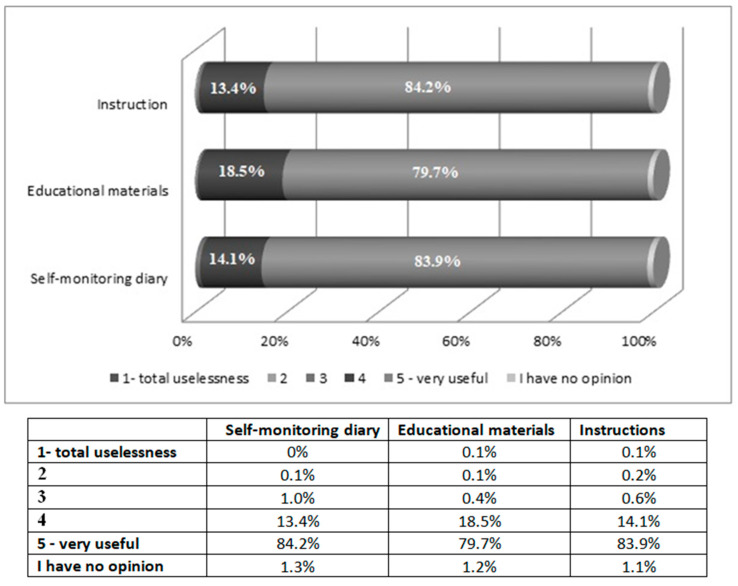
Assessment of education materials.

At the telephone contact following 7 days from the enrollment into the study, patients were asked if they needed any additional information related to the GensuPen use and if they had had to contact the help-line concerning problems using the GensuPen. At the telephone contact following 4 weeks, there was a questionnaire-based interview performed to assess the safety (the number of severe hypoglycemic episodes or adverse events related to GensuPen use) and patients’ comfort with the use of the GensuPen (the questionnaire evaluating the GensuPen and the questionnaire comparing a previous pen injector, if applicable, are presented in Table 1 and Table 2, respectively). Patients assessed the education of proper insulin use received during the site visit while the insulin treatment was initiated (Figure 1).

An adverse event (AE) was defined as any adverse medical occurrence in a patient using a device. Serious AEs were defined as events requiring hospitalization, other than severe hypoglycemia, or was life-threatening. Severe hypoglycemia was defined as an episode of low blood glucose concentration requiring a third-party aid, resulting in a resolution of the symptoms, with or without measuring the plasma glucose concentration. An independent agency conducted a telephone visit, maintaining anonymity and independence from the diabetologist and the sponsor, to encourage the participants to respond without fear of negative impacts on their future care.

The study’s primary objective was to assess the safety and patients’ treatment comfort with the use of the GensuPen, assessed with the use of the questionnaire interviews. The questionnaire was self-prepared and approved by two independent experts in the diabetology field and was pre-tested on a group of 60 randomly selected patients. The questionnaires included questions assessing features of the pens, which were related to the ease of use, utility, convenience, and confidence in correct insulin dose administration. Patients were also asked to state whether they had any problems using the GensuPen and, if so, they were asked to describe them.

The statistical analysis was performed using Statistica v12 (PL, Statsoft, Tulsa, OK, USA) software. For quantitative variables, normality of distribution was tested by the Shapiro–Wilk test. The distribution of a quantitative variable (age) was evaluated based on the mean and standard deviation, while the distribution of categorical variables was presented using percentages. The study was performed following the Declaration of Helsinki. According to the written opinion of the local bioethics committee (KNW/0022/KB/32/11), the study did not require obtaining bioethics committee approval due to its observational nature. All participants gave written informed consent to participate in the study. The manuscript was prepared according to STROBE guidelines [16].

## 3. Results

**Figure 2:**
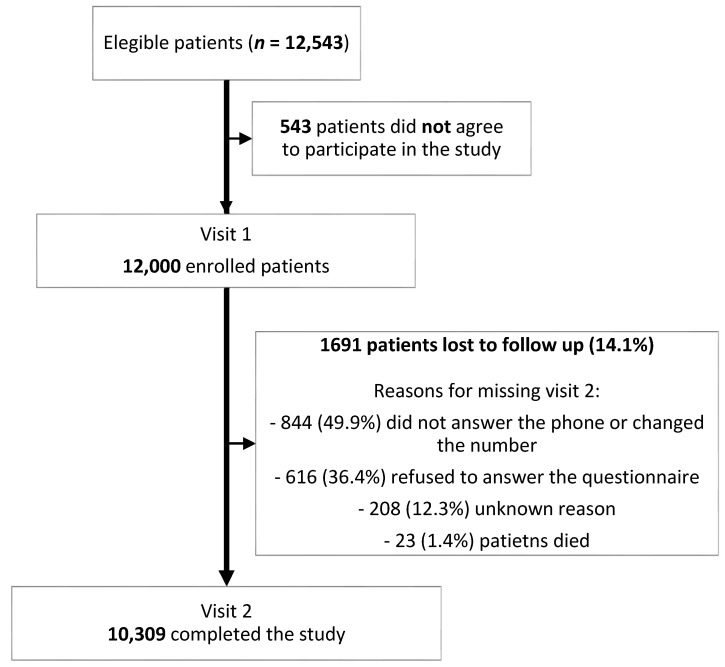
Reason for non-completion.

A total of 12,543 patients were eligible for this study. Twelve-thousand agreed to participate in it and were enrolled, of whom 1691 (14.1%) were excluded from the final safety and comfort analysis due to various reasons (non-completers). The numbers of non-completers, along with the reasons for non-completion, are presented in Figure 2.

The final total sample comprised 10,309 T2DM patients (47.9% women), with a mean age of 63.3 ± 12.0 years. Moreover, 16.0% (*n* = 1649) of patients showed visual impairment and 8.2% (*n* = 845) had impaired manual dexterity.

Most (57.6%, *n* = 5933) of the patients were recruited from the diabetology ward (mean age: 63.3 ± 12.4 years), and the remaining 42.4% (*n* = 4376) were recruited from the outpatient diabetology clinic (mean age: 63.2 ± 11.3 years). For 97.5% (*n* = 10,051) of patients, the GensuPen was their first insulin delivery device used, and 2.5% (*n* = 258) had previously used another insulin delivery device. Among the 2.5% (*n* = 258) of patients who previously used insulin via reusable pens, 17% (*n* = 44) complained about the previous pen injector. A list of complaints regarding the previous pen injector is presented in Table 1. The patients’ assessments of the GensuPen are summarized in Table 2.

Overall, patients rated the GensuPen as very good. They rated it in relation to adjusting the dose forward and backward, correct insulin administration information, trigger location, and force needed for insulin administration. Among the 258 patients who previously used another insulin delivery device, 34.1% (*n* = 88) assessed the sound at setting the dose forward and backward in the GensuPen as better, 56.6% (*n* = 146) as the same, and 9.3% (*n* = 24) as worse. Information indicating that the delivery of insulin administration has ended correctly was assessed in GensuPen as better for 79.8% (*n* = 206) of patients, the same for 12.8% (*n* = 33), and worse for 7.5% (*n* = 19). In the case of trigger location, 26.4% (*n* = 68) of patients reported that the GensuPen was better for insulin injection, 27.1% (*n* = 70) that it was the same, and 46.5% (*n* = 120) that it was worse. Worse assessment of trigger location was driven mainly by patients recruited from hospital wards. Half (49.6%, *n* = 128) of the patients reported that they needed less force to inject insulin; for 32.6% (n = 84), the force was the same, and 7.4% (*n* = 19) had to use more force (Table 3).

Severe hypoglycemia episodes occurred in 0.2% (*n* = 25) of the whole studied group of 10,309 patients. Just 0.6% (*n* = 65) of patients reported adverse events other than hypoglycemia during four weeks of observation. The list of AEs is presented in Table 4. None of the AEs were related solely to GensuPen use. Education performed during the site visit was assessed at the first telephone contact as good enough for 97.2% (*n* = 10,020) of patients, and patients rated the education materials as very good (Figure 1). The vast majority (97.3%, *n* = 10,031) of patients did not need to contact the help-line, and 2.7% (*n* = 278) required more information regarding the technical use of the GensuPen and contacted the help-line.

## 4. Discussion

The presented real-life observational study showed that, in a large group of patients with T2DM, the use of GensuPen is perceived as safe, and patients assess it as comfortable. The majority of study participants rated the GensuPen as very good regarding the setting of the dose forward and backward, correct insulin administration information, trigger location, and force needed for insulin administration. It should be emphasized that the studied patients were mainly elderly, and 16.0% (*n* = 1649) and 8.2% (*n* = 845) of them declared vision disabilities and reduced manual dexterity, respectively. Furthermore, when the features of the GensuPen were compared to the previously used pen injector, it was proven that most of its features (force needed to inject insulin, an indication of insulin correct administration, and audible clicks) were assessed as helpful. Only the trigger location was assessed as worse by 120 patients (1.2% of study participants), and mainly among patients in hospital wards. This phenomenon is difficult to explain. The striking information coming from open questions related to the previous pen is that 44% of patients who previously used another insulin delivery device declared they did not know how to properly administer it. This emphasizes the role of diabetes education and the centrality of the person with diabetes and the “individualization of diabetes self-management education” [17]. The education related to the new insulin pen dispensed to the patient is of utmost importance. The majority of patients reported that obtained education was sufficient, and only 2.7% (*n* = 278) needed to contact the help-line with issues related to the technical use of the GensuPen. The obtained training was in-person, one-on-one, and patients received training materials in writing; the help-line was also toll-free number. However, it should be kept in mind that the observation follow-up was relatively short, and diabetes education is a continuous process, so in real-life, patients should have this repeated over time. Another important issue is safety, where hypoglycemia, especially severe hypoglycemia, is the major concern among patients treated with insulin, because it can be lethal. It is difficult to estimate the exact incidence of hypoglycemia in the elderly due to the low number of studies that enroll older patients and group heterogeneity [18]. There is no doubt that older adults with diabetes possess a greater risk of hypoglycemia and are at risk of worse consequences than younger patients [19]. In this context, in the large group of adult and elderly patients, there were only 0.2% of severe hypoglycemia incidents. In relation to safety, there was a low percentage of AE reported (only 0.6%), and neither was serious. Another issue is the ease of use and comfort, especially for those with impaired vision or reduced manual dexterity, because this can influence the patient’s adherence and efficacy. GensuPen has a large clear scale, which returns to zero to allow visual confirmation of dose delivery; also, the green light indicates that the insulin administration process ended successfully, and patients can hear the click when setting the doses so they can count audible clicks to confirm the dose, which is especially important for patients with vision impairment. According to evidence-based medicine, clinicians need to implement prescribing decisions and patient management based on well-designed studies, preferably randomized controlled trials (RCT). Although this study is observational, data from observational studies have become an increasingly important source of evidence because they reflect the local standards of medical care, have very simple inclusion/exclusion criteria, though observing a heterogeneous population reflecting everyday clinical settings [20,21].

There are several limitations to this study that should be addressed. First, the observational character of the study and lack of active comparator limits the evaluation of the device. Secondly, as with an industry-sponsored trial, an obvious limitation is the manufacturer’s involvement in the funding process. However, it should be noted that most of the published literature on insulin pen devices has been funded by the various manufacturers [11,12,13,14], so the interpretation of the results needs to be done with caution. There was no information regarding why the pen injector was changed, nor for the insulin therapy treatment model, the type of insulin, or the dose of insulin used. We can only suppose that the pen injector was switched to GensuPen either due to complaints related to the previously used pen injector itself or insulin-type issues.

Additionally, hypoglycemia reporting was based solely on the patients’ ability to recall the event, introducing bias. Moreover, potential recall bias related to the comparison of the previously used pens with the GensuPen must be acknowledged, and the fact that the questionnaires used were not validated. Hypoglycemia occurrence and adverse reactions also might be related to the pen injector and the insulin itself. Despite the limitations above, the data is clinically relevant because it provides information related to a heterogeneous real-life local setting and proves the safety and patients’ satisfaction with the GensuPen in a large group of adult and elderly patients.

## 5. Conclusions

With its large size, this observational study provides evidence that, after proper training, which proved to be easy to understand, insulin devices can be easily introduced to adult and elderly patients with T2DM and can be linked to safety and patient comfort. Given the constantly developing diabetes industry where new insulins and new medical devices emerge, it is important to assess utility in a real-life setting, not under strict RCT control, especially among elderly people who may not be able to cope with new technologies.

## Figures and Tables

**Table 1:** Complaints related to previously used insulin pen injectors.

Complaint	Number of Patients *n* = 258 (2.5%)
The insulin injection was too fast or too slow	2.3%
The set dose was higher than intended	0.7%
The wrong dose was delivered	2.1%
The patient did not know the insulin injection technique	44%
Difficult to operate	0.04%
Pen got stuck	0.04%
Needles were getting broken	0.16%
Too heavy	0.08%
Too small	0.04%
Too lose in setting the dose	0.08%
Leaky case	0.19%
The number on the scale too small to read	0.08%
Numbers on the scale clashed	2.8%
Broken case	0.4%
Broken spring	0.2%
Problems with correcting the dose	0.2%
Cartridge too small	0.04%
Left marks on the skin	0.04%
The sheath did not open properly	0.04%

**Table 2:** The questionnaire surveyed at telephone contact assessing the GensuPen use among all study participants (*n* = 10,309).

Question Rate	Very Good	Good	Sufficient	Poor	Very Poor
Setting and correcting the dose	87.8%	10.0%	1.7%	0.3%	0.2%
Information confirming that the dose was administrated successfully	92.0%	4.7%	1.3%	0.6%	1.4%
Trigger location	80.9%	11.6%	2.2%	2.0%	3.3%
Force needed to insulin administration	75.0%	17.2%	3.1%	2.1%	2.6%

**Table 3:** Questionnaire surveyed at telephone contact comparing the GensuPen to the a previously used pen (*n* = 258).

Question Rate	Better	The Same	Worse
Setting and correcting the dose	34.1%	56.5%	9.4%
Information confirming that the dose was administrated correctly	79.8%	12.7%	7.5%
Trigger location	26.3%	27.2%	46.5%
Force needed to insulin administration	50.0%	32.7%	17.3%

**Table 4:** Adverse events occurring during a 4-week follow-up.

Adverse Event	Number of Patients *n* = 65 (0.6%)
Headache	3.2%
Tremor	17.7%
Difficulty breathing	4.8%
Tingle of hands and feet	4.8%
Weakness	9.7%
Swelling	1.6%
Sweating	14.5%
Somnolence	1.6%
High blood pressure	1.6%
Feeling bad	4.8%
Dizziness	8.1%
Fingernails changed color	1.6%
Palpitation	1.6%
